# Up regulation and nuclear translocation of Y-box binding protein 1 (YB-1) is linked to poor prognosis in ERG-negative prostate cancer

**DOI:** 10.1038/s41598-017-02279-x

**Published:** 2017-05-17

**Authors:** Asmus Heumann, Özge Kaya, Christoph Burdelski, Claudia Hube-Magg, Martina Kluth, Dagmar S. Lang, Ronald Simon, Burkhard Beyer, Imke Thederan, Guido Sauter, Jakob R. Izbicki, Andreas M. Luebke, Andrea Hinsch, Frank Jacobsen, Corinna Wittmer, Franziska Büscheck, Doris Höflmayer, Sarah Minner, Maria Christina Tsourlakis, Thorsten Schlomm, Waldemar Wilczak

**Affiliations:** 1 Institute of Pathology, University Medical Centre Hamburg-Eppendorf, Hamburg, Germany; 2 General, Visceral and Thoracic Surgery Department and Clinic, University Medical Centre Hamburg-Eppendorf, Hamburg, Germany; 3 Martini-Clinic, Prostate Cancer Centre, University Medical Centre Hamburg- Eppendorf, Hamburg, Germany; 4 Department of Urology, Section for translational Prostate Cancer Research, University Medical Centre Hamburg-Eppendorf, Hamburg, Germany

## Abstract

Y-box binding protein 1 (YB-1) is an RNA and DNA binding factor with potential prognostic cancer. To evaluate the clinical impact of YB-1, a tissue microarray with 11,152 prostate cancers was analysed by immunohistochemistry. Cytoplasmic and nuclear staining was separately analysed. Cytoplasmic YB-1 was absent or weak in normal epithelium but seen in 86,3% of carcinomas. Cytoplasmic staining was weak, moderate, and strong in 29.6%, 43.7% and 13.0% of tumours and was accompanied by nuclear YB-1 staining in 32.1% of cases. Particularly nuclear staining was strongly linked to poor patient prognosis (p < 0.0001). YB-1 protein was more abundant in ERG positive (95.1%) than in ERG negative cancers (80.4%; p < 0.0001), but any prognostic impact of YB-1 staining was limited to the ERG-negative subset. Similarly, significant associations with pT stage and Gleason grade (p < 0.0001 each) were driven by the ERG negative subset. The significant association of YB-1 protein detection with deletions of *PTEN*, *5q21 and 6q15* fits well in the protein’s role as an inhibitor of DNA damage dependent cell cycle arrest, a role that is likely to induce genomic instability. In summary, the data show, that the prognostic impact of YB-1 expression is limited to ERG negative prostate cancers.

## Introduction

Prostate cancer is the most prevalent cancer in men in Western societies^1^. Although the majority of prostate cancers behave in an indolent manner, a small subset is highly aggressive and requires active treatment^2, 3^. Established preoperative prognostic parameters are limited to Gleason grade and tumour extent on biopsies, prostate-specific antigen (PSA) as well as clinical stage. These data are statistically powerful, but often insufficient for optimal treatment decisions in individual patients. It is, thus, hoped that a better understanding of disease biology will eventually lead to the identification of clinically applicable molecular markers that enable a more reliable prediction of prostate cancer aggressiveness.

Y-box binding protein 1 (YB-1) is a DNA- and RNA-binding protein and transcription factor with an evolutionarily ancient and conserved cold shock domain^4^, that is involved in multiple cellular processes. YB-1 is a major component of protein complexes mediating mRNA binding and transport^5^. In response to genotoxic stress YB-1 translocate from the cytoplasm to the nucleus^6^, where it acts as a transcriptional regulator to overcome DNA damage dependent cell cycle arrest and to promote cell survival^5^. Given the “oncogenic” nature of these functions, it is not unexpected that up-regulation of YB-1 has been reported in virtually all human cancer types, including epithelial and mesenchymal solid cancers^7–16^ as well as haematological diseases like lymphoma and leukaemia^17, 18^. YB-1 overexpression has been linked to adverse clinical outcome and poor therapy response in most of them^7–12, 14, 15, 17, 18^, making it a promising prognostic biomarker in cancer. YB-1 may also play an important role in prostate cancer, since studies on prostate cancer cell lines and clinical specimens suggest that it may be implicated in androgen receptor (AR) signalling and resistance to anti-androgenic therapy^6, 19, 20^. Previous work on 35–380 prostate cancers has suggested that YB-1 up-regulation may be linked to development and progression of this malignancy^6, 20–24^.

To better understand the prognostic value of YB-1 expression in prostate cancer, we took advantage of our existing large prostate cancer tissue microarray with its attached database containing histological, clinical, and molecular data from more than 11,000 patients. The results of our immunohistochemical analyses demonstrate that YB-1 has clinically relevant prognostic value, particularly in prostate cancers lacking the *TMPRSS2*:*ERG* fusion gene.

## Results

### Technical issues

A total of 6,935 (62.2%) of tumour samples were interpretable in our TMA analysis. Reasons for non-informative cases (4,230, 37.5%) included lack of tissue spots or absence of unequivocal cancer tissue in the TMA spot.

### YB-1 expression in normal and cancerous prostate tissue

**Figure 1:**
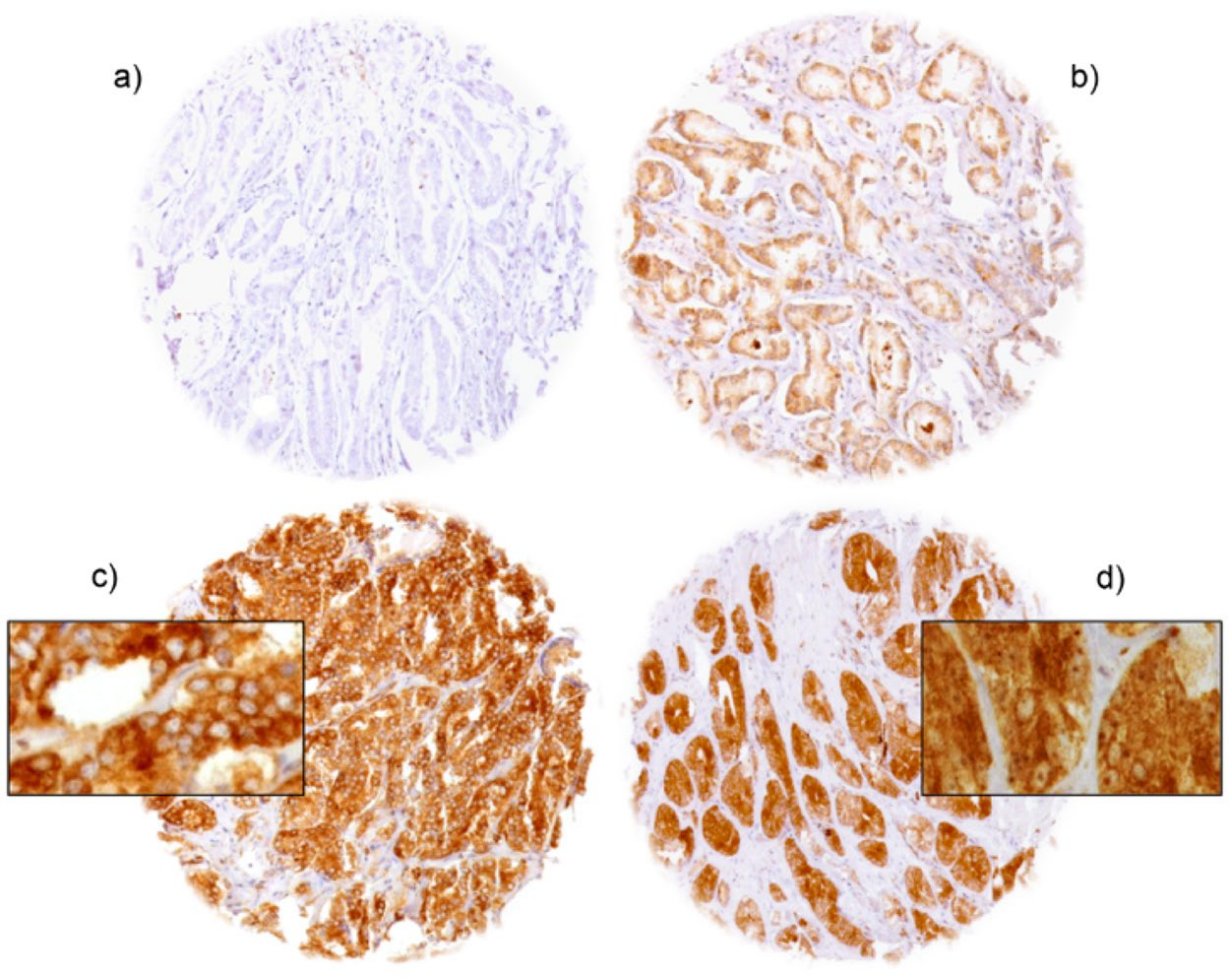
Representative pictures of YB-1 staining in prostate cancer with (**a**) negative, (**b**) moderate, (**c**) strong cytoplasmic staining and (**d**) nuclear accumulation.

Normal prostate epithelium showed either none or weak cytoplasmic staining but always lacked nuclear YB-1 positivity in epithelial and stromal cells. In cancers, cytoplasmic YB-1 staining was typically more intense as compared to normal prostate glands. Representative images of YB-1 staining are given in Fig. 1. Cytoplasmic staining was seen in 5,984 of our 6,935 (86.3%) interpretable prostate cancers and was considered weak in 29.6%, moderate in 43.7% and strong in 13.0% of cases (Table 1). Positive cytoplasmic staining was accompanied by nuclear staining in 2,015 (33.7%) of 5,984 cases. Unequivocal nuclear staining was never seen in the absence of concomitant cytoplasmic staining. To better understand the individual impact of cytoplasmic and nuclear staining, we grouped all cancers according to (1) the level of cytoplasmic staining (regardless of nuclear staining) and (2) the co-expression patterns of cytoplasmic and nuclear staining according to the following criteria: no staining at all (negative), cytoplasmic staining without nuclear co-staining (cytoplasmic only), cytoplasmic staining with nuclear co-staining (nuclear accumulation).

**Table 1:** Association between YB-1 staining results and prostate cancer phenotype.

Parameter	N evaluable	Cytoplasmic YB-1 staining (%)				P value	Nuclear accumulation (%)	P value
		Negative	Weak	Moderate	Strong			
**All cancers**	6,935	14.2	29.6	43.3	13.0		29.1	
**Tumour stage**						0.0165		<0.0001
pT2	4,359	14.9	29.1	43.4	12.7		26.6	
pT3a	1,663	11.6	30.7	43.8	13.9		33.1	
pT3b-pT4	887	11.8	30.6	44.5	13.1		37.0	
**Gleason grade**						<0.0001		<0.0001
≤3 + 3	1,523	19.9	28.3	39.7	12.1		20.2	
3 + 4	3,567	12.1	30.4	44.4	13.1		30.4	
3 + 4 tertiary 5	296	15.3	25.8	46.8	12.1		41.5	
4 + 3	603	11.1	26.0	47.1	15.8		33.9	
4 + 3 tertiary 5	424	9.3	29.2	48.2	13.3		41.9	
≥4 + 4	238	11.8	31.9	44.1	12.2		43.8	
**Lymph node metastasis**						0.8530		0.0056
N0	3,974	12.8	30.2	43.9	13.1		30.4	
N+	364	12.9	31.9	43.4	11.8		38.5	
**Preoperative PSA level (ng/ml)**						<0.0001		0.008
<4	803	11.5	29.3	44.7	14.6		30.1	
4–10	4,165	13.0	28.7	44.3	13.9		28.9	
10–20	1,384	15.5	30.9	43.1	10.5		30.0	
>20	499	18.2	34.5	38.1	9.2		31.8	
**Surgical margin**						0.1780		0.5781
Negative	5,481	13.7	29.2	43.7	13.4		29.2	
Positive	1,338	13.2	31.8	43.2	11.7		30.5	

### Associations with tumour phenotype

Cytoplasmic and nuclear YB-1 staining showed different associations with tumour phenotype. Nuclear YB-1 accumulation was significantly linked to advanced tumour stage and high Gleason grade and patient age (p < 0.0001 each) and to a lesser extent to nodal status (p = 0.0056; Table 1). In contrast, cytoplasmic YB-1 expression levels differed much less between tumours with varying grades and stages although significant p values were still found in some categories due to the high numbers of analysed samples (Table 1). Subset analyses of ERG-negative and ERG-positive cancers showed, that all these associations were somewhat stronger in the subset of ERG negative cancers (Supplementary Table S1) as compared to ERG-positive cancers (Supplementary Table S2).

### Association with *TMPRSS2*:*ERG* fusion status and ERG protein expression

**Figure 2:**
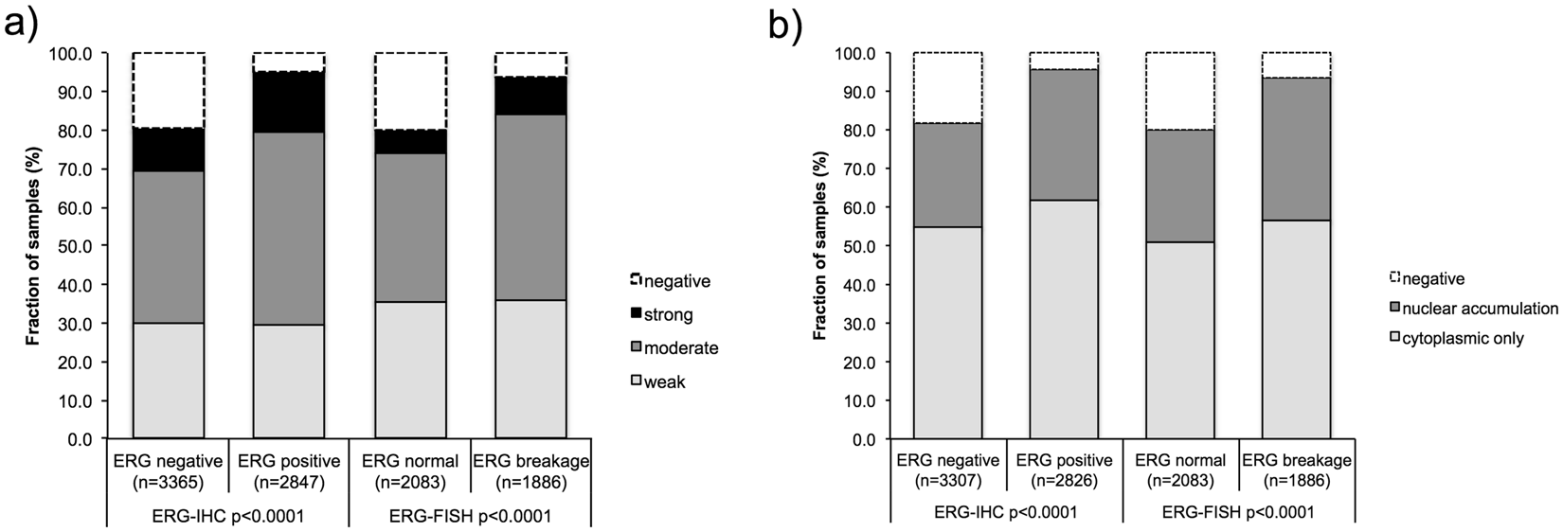
Association between positive YB-1 staining and TMPRSS2:ERG- fusion status as measured by immunohistochemistry (IHC) and fluorescent *in situ* hybridization (FISH) (**a**) cytoplasmic YB-1 staining, (**b**) nuclear YB-1 accumulation.

Data on both ERG FISH and IHC were available from 3,823 cancers with evaluable YB-1 staining, and an identical result (ERG IHC negative and missing break by FISH or ERG IHC positive and break by FISH) was found in 96% and 95.6% of the examined cancers. Both cytoplasmic YB-1 expression and nuclear accumulation were linked to *TMPRSS2*:*ERG* rearrangement and ERG expression (Fig. 2; p < 0,0001 each). For example, positive cytoplasmic YB-1 staining was seen in 80.4% (including 27% cases with nuclear co-staining) of ERG-IHC negative but in 95.1% (34.2% nuclear co-staining) of ERG-IHC positive cancers (p ≤ 0.05 each).

### Association to key genomic deletions

**Figure 3:**
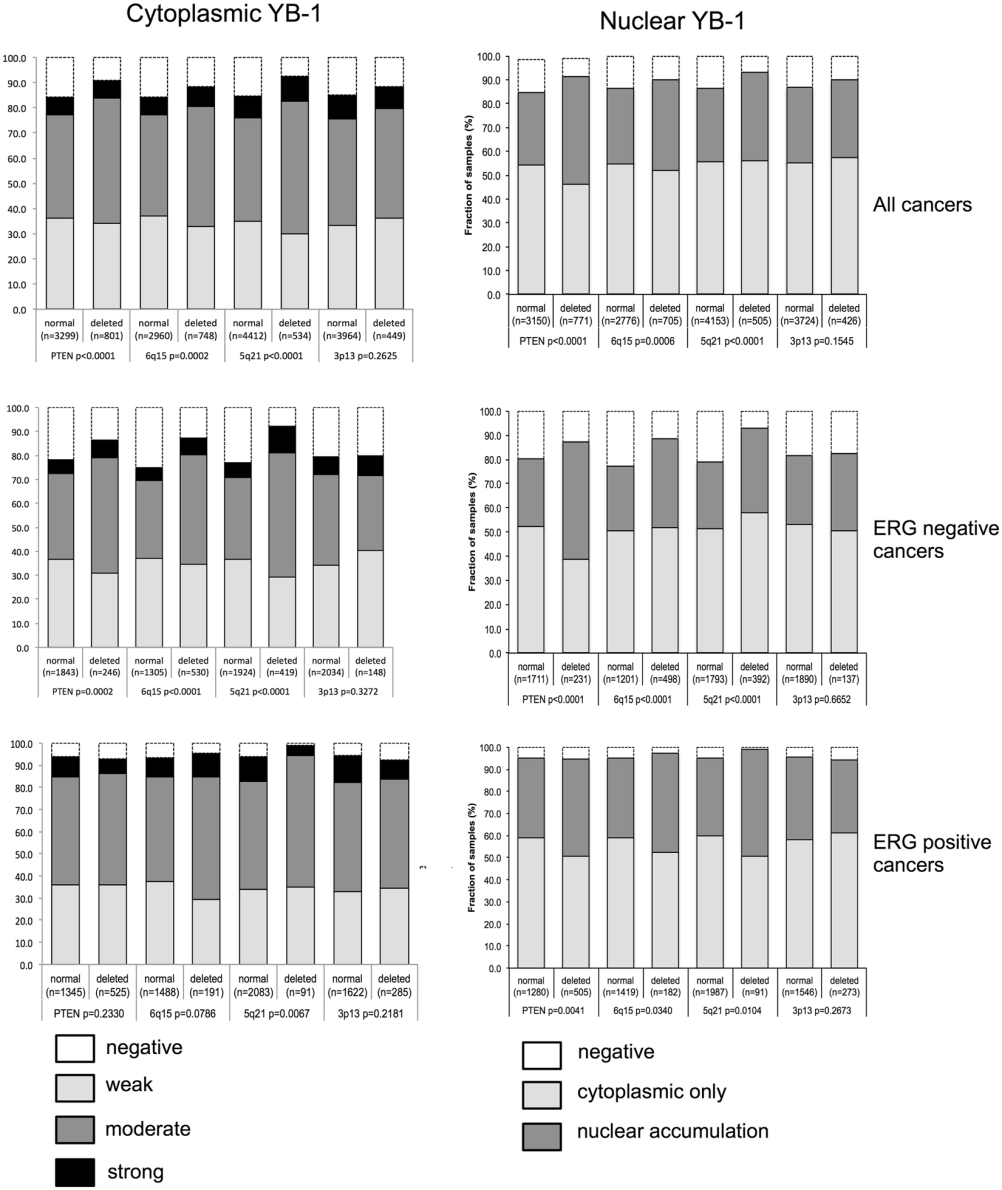
Association between YB-1 staining and deletion of 10q23 (PTEN), 5q21 (CHD1), 6q15 (MAP3K*7*), 3p13 (FOXP1) in all cancers, the ERG-negative and ERG-positive subset.

Comparison of YB-1 expression with several of the most frequent genomic deletions (*PTEN*, 3p13, 6q15. and 5q21) revealed that both cytoplasmic and nuclear YB-1 staining was significantly linked to deletions of *PTEN* (10q32), 5q21 (*CHD1*) and 6q15 (*MAP3K7*; p ≤ 0.0006 each, Fig. 3). Subset analyses of ERG-positive and ERG-negative cancers revealed that these associations were largely driven by the subset of ERG-negative cancers (p < 0.0001 each, Fig. 3).

### Associations with PSA recurrence

**Figure 4:**
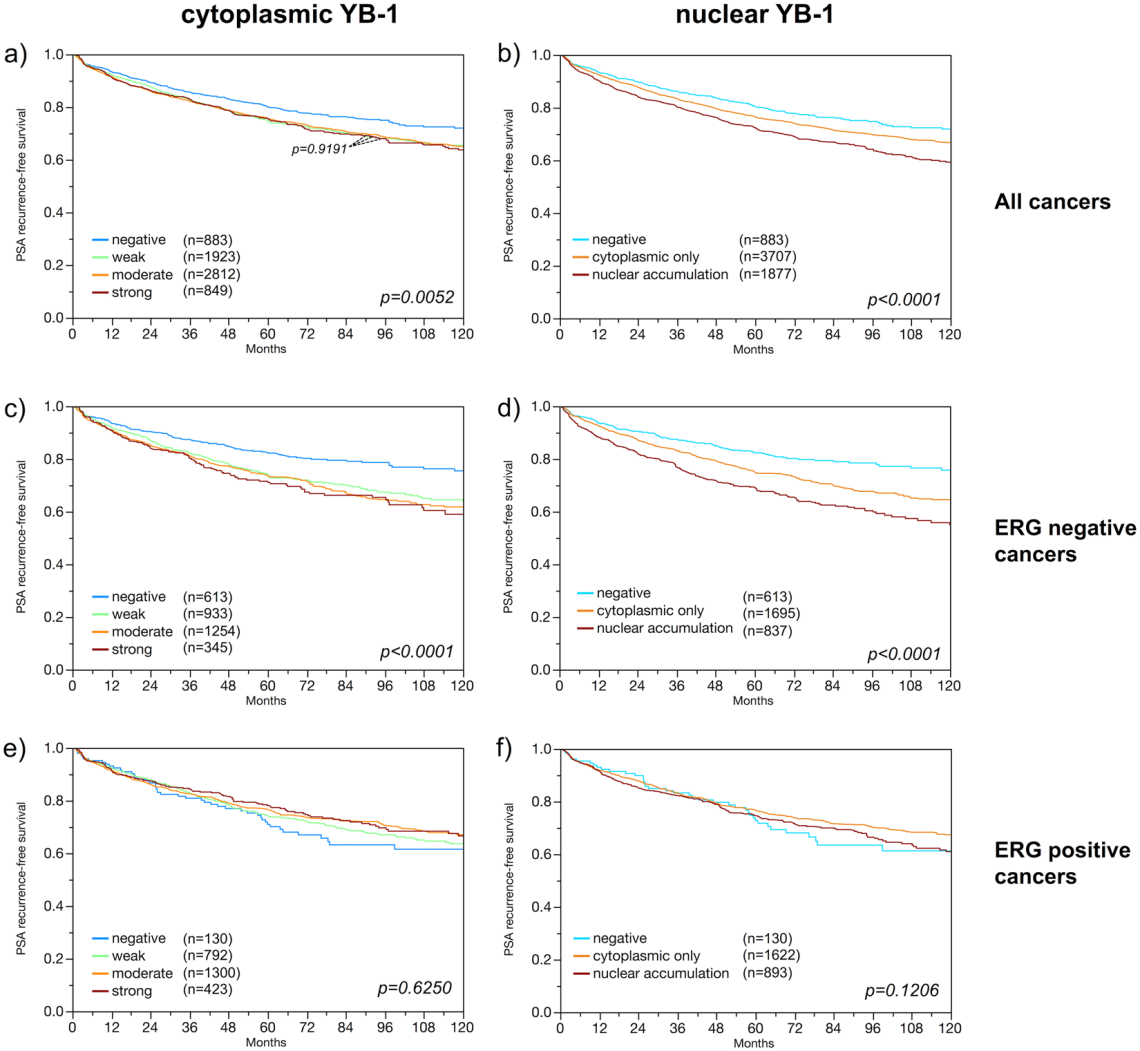
Prognostic impact on prostate specific antigen (PSA) recurrence after radical prostatectomy of cytoplasmic YB-1 expression and nuclear YB-1 accumulation in all cancers (**a**,**b**), ERG fusion positive cancers (**c**,**d**) and ERG fusion negative cancers (**e**,**f**).

Follow-up data were available for 6,353 patients with interpretable YB-1 staining on the TMA. pT stage (Table 2), traditional Gleason grade (Supplementary Fig. S1a), and quantitative Gleason grade (Supplementary Fig. S1b–h) were strongly associated with PSA recurrence. Presence of already minimal quantities of cytoplasmic YB-1 staining was strongly linked to early biochemical recurrence (p < 0.0001), but increasing staining levels did not lead to a further deterioration of the prognosis (p = 0.9191, Fig. 4a). Factoring in the staining localization revealed that the prognosis of YB-1 positive cancers was particularly poor if nuclear YB-1 staining was seen (p < 0.0001, Fig. 4b). Further analyses in ERG-negative and ERG-positive cancers revealed that the prognostic impact of both cytoplasmic and nuclear YB-1 staining was strictly limited to the subset of ERG-negative cancers (p < 0.0001 each for cytoplasmic and combined cytoplasmic/nuclear staining, Fig. 4c,d) but not seen in ERG-positive cancers (Fig. 4e,f). To better understand the prognostic power of nuclear YB-1 accumulation in ERG-negative cancers, we performed further subset analyses in cancers with identical classical and quantitative Gleason scores. Nuclear localization of YB-1 staining did not provide significant prognostic information in any subsets defined by the classical Gleason score (Supplementary Fig. S1a), nor by the quantitative Gleason score (Supplementary Fig. S1b–h).

**Table 2:** Pathological and clinical data of the arrayed prostate cancers.

	No. of patients (%)	
	Study cohort on TMA	Biochemical relapse among categories
	(N = 11,152)	(N = 1,824; 18.5%)
**Follow-up (month)**		
Mean	60	—
Median	65.6	—
**Age (y)**		
≥50	323	51 (15.8%)
51–59	2696	445 (16.5%)
60–69	6528	1078 (16.5%)
≥70	1498	241 (16.1%)
**Pre-treatment PSA (ng/ml)**		
<4	1417	142 (10.0%)
4–10	6866	823 (12.0%)
10–20	2160	525 (24.3%)
>20	719	308 (42.8%)
**pT category (AJCC 2002)**		
pT2	7514	565 (7.5%)
pT3a	2403	586 (24.4%)
pT3b	1265	623 (49.2%)
pT4	63	49 (77.8%)
**Gleason grade**		
≤3 + 3	2734	342 (12.5%)
3 + 4	5622	1057 (18.8%)
3 + 4 tertiary 5	379	84 (22.2%)
4 + 3	912	405 (44.4%)
4 + 3 tertiary 5	520	230 (44.2%)
≥4 + 4	416	221 (53.1%)
**pN category**		
pN0	6115	1126 (18.4%)
pN+	568	298 (52.5%)
**Surgical margin**		
Negative	8999	1148 (12.8%)
Positive	2096	639 (30.5%)

NOTE: Numbers do not always add up to 11,152 in the different categories because of cases with missing data. Percentage in column “Biochemical relapse among categories” refers to the fraction of samples with biochemical relapse within each parameter in the different categories. Abbreviation: American Joint Committee on Cancer (AJCC).

### Multivariate analysis

Four different types of multivariate analyses were performed evaluating the clinical relevance of nuclear YB-1 accumulation in different scenarios in the subset of ERG-negative cancers (Table 3). Scenario 1 evaluated all postoperatively available parameters (pathological tumour stage, pathological lymph node status (pN), surgical margin status, preoperative PSA value and pathological Gleason grade obtained after the morphological evaluation of the entire resected prostate). Scenario 2 is identical to scenario 1 but without nodal status. The rational for this approach was that the indication and extent of lymph node dissection is not standardized in radical prostatectomy and that excluding pN in multivariate analysis can markedly increase case numbers. Two additional scenarios had the purpose to model the preoperative situation as much as possible. Scenario 3 included YB-1 expression, preoperative PSA, clinical tumour stage (cT stage) and Gleason grade obtained on the prostatectomy specimen. Since postoperative determination of a tumour’s Gleason grade is “better” than the preoperatively determined Gleason grade (subjected to sampling errors and consequently under-grading in more than one third of cases)^25^, another multivariate analysis was added. In scenario 4, the preoperative Gleason grade obtained on the original biopsy was combined with preoperative PSA, cT stage and YB-1 expression. Nuclear YB-1 expression provided significant independent prognostic value beyond the established parameters in all scenarios if the analysis was limited to ERG-negative cancers.

**Table 3:** Multivariate analysis of nuclear accumulation of YB-1 in ERG-negative cancers in different clinical scenarios.

Scenario	N analyzable	P value							
		Preoperative PSA-Level	pT Stage	cT Stage	Gleason grade prostatectomy	Gleason grade biopsy	pN Stage	R Status	YB1 nuclear accumulation
1	3,153	0.0066	<0.0001	—	<0.0001	—	<0.0001	0.0019	0.0347
2	3,156	0.0005	<0.0001	—	<0.0001	—	—	0.0006	0.0427
3	3,077	<0.0001	—	<0.0001	<0.0001	—	—	—	0.0106
4	3,033	<0.0001	—	<0.0001		<0.0001		—	0.0001

## Discussion

The results of our study demonstrate that nuclear YB-1 accumulation is a strong and independent predictor of early biochemical recurrence in the subset of prostate cancers lacking ERG-fusion.

YB-1 staining was found in the cytoplasm and often also in the nuclei of cancer cells, but was typically weaker and limited to the cytoplasmic compartment in normal prostate glands. These findings suggest a general role of YB-1 up-regulation for prostate cancer biology, and a particular importance of nuclear YB-1 accumulation. Of note, the lack of nuclear YB1 staining in normal glands and in a fraction of our cancers does not mean that YB-1 is absent or post-transcriptionally modified in these nuclei, but rather that its quantity is below the detection threshold of our experimental conditions. As YB-1 is a ubiquitous protein that is present in virtually all human cell types^26^ our immunohistochemistry protocol was selected to distinguish tissues with low (i.e., expression level below the IHC detection threshold) and high expression (i.e., very strong staining). It is thus not surprising, that nuclear staining was found in normal prostate glands and in stromal cells of normal prostate parenchyma in an earlier study probably using a more sensitive protocol^20^. Nevertheless, our finding of a cytoplasmic staining in >85% and nuclear staining in about 30% of cancers is in line with earlier studies reporting moderate to strong cytoplasmic staining in 63% and 71% of tumours, and strong nuclear staining in 20% and 34% tumours in cohorts of 35 and 165 prostate cancers^20, 23^.

A comparison of our IHC data with PSA recurrence revealed that – irrespective of the staining intensity - presence of detectable YB-1 protein was associated with unfavourable prognosis and that the risk for early PSA recurrence further increased if nuclear staining was also seen. This observation is in line with different functions of nuclear and cytoplasmic YB-1. In the cytoplasm, YB-1 is a major component of messenger Ribonucleoproteins (mRNPs), which bind to and regulate translation of mRNA. It has been shown that YB-1 translocate from the cytoplasm to the nucleus in response to genotoxic stress^6^. In the nucleus, YB-1 functions as a pro-survival factor, which initiates transcriptional programs associated with control of DNA repair, cell proliferation and inhibition of apoptosis^5^. These are key features of malignant transformation. The particular prognostic role of nuclear YB-1 accumulation is thus not unexpected. Our findings are in line with several earlier findings. One study on 165 patients reported, that YB-1 accumulated in the nucleus after activation by phosphorylation at Serine 102, and found that nuclear phospho-YB-1^Ser102^ was more linked to early PSA recurrence than cytoplasmic YB-1^23^. Two other studies described associations between nuclear YB-1 staining and high Gleason grades in cohorts of 35 and 366 prostate cancers^6, 20^. Another study on 332 prostate cancers described mainly cytoplasmic YB-1 staining to be associated with a reduced 5 year PSA recurrence-free survival^21^. Finding an association with patient age is interesting in the light of earlier data suggesting age-dependent changes of YB-1 levels in mouse models^27^.

The large number of cancers on our TMA and the molecular database attached to it allowed us to draw some conclusions on the molecular mechanisms associated with YB-1 up-regulation “in silico”. These data revealed, that both cytoplasmic and nuclear YB-1 levels were higher in ERG-positive than in ERG-negative cancers. Finding this association by two independent approaches for ERG fusion detection (IHC/FISH) largely excludes a false positive association due to inefficient staining for YB-1 and ERG in a subset of damaged non-reactive tissues. ERG activation is the most frequent molecular alteration in prostate cancer. More than half of all prostate cancers, particularly those of young patients, carry gene fusions linking the androgen-regulated *TMPRSS2* gene with the transcription factor *ERG* ^28, 29^. These genomic rearrangements result in an androgen-driven overexpression of ERG in affected cells^30^ and, thus, altered expression of more than 1,600 genes in prostate epithelial cells^31^. The exact nature of an ERG/YB-1 interaction remains elusive. There are, however, potential indirect interactions. For example, YB-1 may affect double strand breakage (DSB) repair through its regulation of Ku80^5^. Ku80 binds to DNA double-strand break ends, where it functions as a molecular scaffold to which other proteins involved in non-homologous end joining can bind^32^. ERG-fusions are chromosomal rearrangements involving transient double strand breakages (DSB)^33, 34^, which are normally removed by specific DSB repair mechanisms^35^. Using the same TMA as in our current study, we earlier found that overexpression of other DSB repair genes such as NBS1^36^ and LIG4^37^ were also linked to ERG-activation in prostate cancer.

Deletions of certain small and large chromosomal regions are another hallmark of prostate cancer. Data from next generation sequencing studies demonstrate that such deletions are more prevalent than any mutations of specific coding genes and many of these deletions have been linked to either ERG-positive (i.e. *PTEN* and 3p13)^38, 39^ or ERG-negative cancers (i.e. 6q15 and 5q23)^40, 41^. Finding a strong link between virtually all of these deletions and YB-1 up-regulation or nuclear accumulation fits well to the concept, that a nuclear protein that suppresses DNA damage dependent cell cycle arrest would induce genetic instability^42^. *In vitro* studies have shown that YB-1 contributes to inhibition of mismatch repair, elevated mutation frequency, mitotic failure, and centrosome amplification^42, 43^. That associations between YB-1 and chromosomal deletions were markedly stronger in the subset of ERG-negative cancers may suggest a diminished impact of YB-1 on the DSB repair machinery in the presence of ERG fusion. Earlier work has indeed demonstrated that ERG can inhibit components of the double strand breakage (DSB) machinery^44^. It is also possible, that the generally higher YB-1 expression levels in ERG-positive than in ERG-negative cancers makes it more difficult to see further differences in expression with the selected experimental conditions.

That YB-1 analysis provided prognostic information independent from established pre- and postsurgical parameters make this protein a promising candidate for routine diagnostic tests. This is all the more true because three distinct prognostic groups could be distinguished simply based on the presence or absence of cytoplasmic and nuclear staining. Such yes/no answers are optimal for diagnostic purposes. The striking limitation of the prognostic relevance of YB-1 to the subset of ERG-negative cancers raises the issue of “subtype specific” prognostic tests. Using the same TMA as in our present study, we have earlier identified several molecular markers with variable prognostic impact depending on the molecular environment. For example, we found that the prognostic value of SOX9 [41] and mTOR expression [42] was limited to ERG-positive cancers while the prognostic value of NBS1 [43], SENP1^45^ CD147^46^ only seen in ERG-negative prostate cancers. These findings challenge the concept of universal prognostic marker sets for all prostate cancers, which have been advertised during the last years^47, 48^. It appears plausible that future tests including molecular subtype specific marker sets will be able to improve patient prognosis prediction.

The Gleason grade is the gold standard for molecular prognostic tests. Besides a reproducibility issue, its main “weakness” is that only 5 prognostic groups are traditionally distinguished, three of which harbour a rather ominous prognosis (Gleason 4 + 3, 8, and 9–10). In a recent study using own data from more than 10,000 patients we had demonstrated, that by using the percentage of unfavourable Gleason patterns, the Gleason grading can be upgraded to an even finer distinction of prognostic subgroups^49^. That the prognostic impact of YB-1 expression largely disappeared in groups defined by conventional Gleason grade categories or by comparable percentages of Gleason 4 patterns demonstrates the power of morphologic malignancy assessment. These findings show that the requirements for a molecular test to be clearly better than tumour morphology are really high.

In summary, our study demonstrates that the prognostic impact of YB-1 in prostate cancer depends on the ERG fusion status and on the intracellular localization of the protein. Detection of nuclear YB-1 may have clinical value to identify a subgroup of patients with aggressive ERG-negative prostate cancers.

## Material and Methods

### Ethical statement

The study was approved by the Ethics commission Hamburg, WF-049/09 and PV3652 and conducted in accordance with the Declaration of Helsinki. Informed consent has not been collected specifically for the patient samples included in this study. Usage of routinely archived formalin fixed leftover patient tissue samples for research purposes by the attending physician is approved by local laws and does not require written consent (HmbKHG, §12,1).

### Patients

Radical prostatectomy specimens were available from 11,156 patients, undergoing surgery between 1992 and 2012 at the Department of Urology and the Martini Clinics at the University Medical Centre Hamburg-Eppendorf. Histo-pathological data was retrieved from the patient files, including tumour stage, Gleason grade, nodal stage and stage of the resection margin. In addition to the classical Gleason categories, “quantitative” Gleason grading was performed as described before^49^. In brief, for every prostatectomy specimen, the percentages of Gleason 3, 4, and 5 patterns were estimated in cancerous tissues during the regular process of Gleason grading. Gleason 3 + 4 and 4 + 3 cancers were subdivided according to their percentage of Gleason 4. For practical use, we subdivided Gleason 3 + 4 and 4 + 3 cancers in 8 subgroups: 3 + 4 ≤ 5% Gleason 4, 3 + 4 6–10%, 3 + 4 11–20%, 3 + 4 21–30%, 3 + 4 31–49%, 4 + 3 50–60%, 4 + 3 61–80% and 4 + 3 > 80% Gleason 4. In addition, separate groups were defined by the presence of a tertiary Gleason 5 pattern, including 3 + 4 tertiary 5 and 4 + 3 tertiary 5. Follow-up data were available for a total of 11,147 patients with a median follow-up of 36 months (range: 1 to 241 months; Table 2). Prostate specific antigen (PSA) values were measured following surgery and PSA recurrence was defined as the time point when postoperative PSA was at least 0.2 ng/ml and increasing at subsequent measurements. All prostate specimens were analysed according to a standardized procedure, including a complete embedding of the entire prostate for histological analysis^50^. The TMA manufacturing process was described earlier in detail^51^. In short, one 0.6 mm core was taken from a representative tissue block from each patient. The tissues were distributed among 27 TMA blocks, each containing 144 to 522 tumour samples. For internal controls, each TMA block also contained various control tissues, including normal prostate tissue. The molecular database attached to this TMA contained results on ERG expression in 10,678^29^, *ERG* break apart FISH analysis in 7,099 (expanded from)^52^ and deletion status of 5q21 (*CHD1*) in 7,932 (expanded from)^41^, 6q15 (*MAP3K7*) in 6,069 (expanded from)^40^, *PTEN* (10q23) in 6,704 (expanded from)^38^, and 3p13 (*FOXP1*) in 7,081 (expanded from)^39^ cancers.

### Immunohistochemistry

Freshly cut TMA sections were immunostained on one day and in one experiment. Slides were deparaffinised and exposed to heat-induced antigen retrieval for 5 minutes in an autoclave at 121 °C in pH 7,8 Tris-EDTA-Citrate buffer. Primary antibody specific for YB-1 (rabbit polyclonal antibody, ab12148, Abcam, Cambridge, UK, dilution 1:450) was applied at 37 °C for 60 minutes. Bound antibody was then visualized using the EnVision Kit (Dako, Glostrup, Denmark) according to the manufacturer’s directions. A pre-absorption control experiment, using a mixture of the primary antibody and YB-1 blocking peptide (Abcam ab12411) in 50-fold excess, was carried out in parallel to the original IHC protocol on a small TMA containing normal and cancerous test tissues to ensure target-specificity of the antibody. An example is given in Supplementary Fig. S2. In prostate tissues, YB-1 staining of variable intensity was seen in the cytoplasm, which was often accompanied by nuclear co-staining of similar intensity. Since YB-1 positive cancers typically showed staining of all (100%) tumor cells, we recorded the cytoplasmic staining intensity (0, 1+, 2+, and 3+) as well as the presence or absence of nuclear co-staining for each tissue spot.

### Statistics

Statistical calculations were performed with JMP® 10.0.2 software (SAS Institute Inc., NC, USA). Contingency tables and the chi²-test were performed to search for associations between molecular parameters and tumour phenotype. Survival curves were calculated according to Kaplan-Meier. The Log-Rank test was applied to detect significant differences between groups. Cox proportional hazards regression analysis was performed to test the statistical independence and significance between pathological, molecular and clinical variables. Separate analyses were performed using different sets of parameters available either before or after prostatectomy. The statistical analysis was performed separately for cytoplasmic and nuclear staining as well as for combined cytoplasmic and nuclear staining.

## Electronic supplementary material

Supplementary Material
