# Author Correction: Up regulation and nuclear translocation of Y-box binding protein 1 (YB-1) is linked to poor prognosis in ERG-negative prostate cancer

**DOI:** 10.1038/s41598-018-30975-9

**Published:** 2018-08-21

**Authors:** Asmus Heumann, Özge Kaya, Christoph Burdelski, Claudia Hube-Magg, Martina Kluth, Dagmar S. Lang, Ronald Simon, Burkhard Beyer, Imke Thederan, Guido Sauter, Jakob R. Izbicki, Andreas M. Luebke, Andrea Hinsch, Frank Jacobsen, Corinna Wittmer, Franziska Büscheck, Doris Höflmayer, Sarah Minner, Maria Christina Tsourlakis, Thorsten Schlomm, Waldemar Wilczak

**Affiliations:** 1 Institute of Pathology, University Medical Centre Hamburg-Eppendorf, Hamburg, Germany; 2 General, Visceral and Thoracic Surgery Department and Clinic, University Medical Centre Hamburg-Eppendorf, Hamburg, Germany; 3 Martini-Clinic, Prostate Cancer Centre, University Medical Centre Hamburg- Eppendorf, Hamburg, Germany; 4 Department of Urology, Section for translational Prostate Cancer Research, University Medical Centre Hamburg-Eppendorf, Hamburg, Germany

Correction to: *Scientific Reports* 10.1038/s41598-017-02279-x, published online 17 May 2017

The Article contains errors.

Reference 12 is cited to support a statement that YB-1 is up-regulated in different cancers, and that YB-1 overexpression is linked to adverse clinical outcomes. However, this reference reports that the results are dependent on the antibody against YB-1. As such the authors do not think its citation in the original text is appropriate and wish to remove it.

As a result, in the Introduction,

“Given the “oncogenic” nature of these functions, it is not unexpected that up-regulation of YB-1 has been reported in virtually all human cancer types, including epithelial and mesenchymal solid cancers^7,8,9,10,11,12,13,14,15,16^ as well as haematological diseases like lymphoma and leukaemia^17,18^. YB-1 overexpression has been linked to adverse clinical outcome and poor therapy response in most of them^7,8,9,10,11,12,14,15,17,18^, making it a promising prognostic biomarker in cancer.”

should read:

“Given the “oncogenic” nature of these functions, it is not unexpected that up-regulation of YB-1 has been reported in virtually all human cancer types, including epithelial and mesenchymal solid cancers^7,8,9,10,11,13,14,15,16^ as well as haematological diseases like lymphoma and leukaemia^17,18^. YB-1 overexpression has been linked to adverse clinical outcome and poor therapy response in most of them^7,8,9,10,11,14,15,17,18^, making it a promising prognostic biomarker in cancer.”

Additionally, the following sentence citing this reference should be included in the Materials and Methods section, under the subheading ‘Immunochemistry’:

“Of note, this antibody has been shown to detect an epitope also present on heterogeneous nuclear ribonucleoprotein A1 (Wooley *et al*.^12^).”

