# Supplementary material for: Up regulation and nuclear translocation of Y-box binding protein 1 (YB-1) is linked to poor prognosis in ERG-negative prostate cancer
Source: Sci Rep. 2017 May 17;7:2056. doi: 10.1038/s41598-017-02279-x (PMC5435682; doi:10.1038/s41598-017-02279-x)
Supplement: Supplementary file 1 — Supplementary Material [file 41598_2017_2279_MOESM1_ESM.doc]

**Up regulation and nuclear translocation of Y-box binding protein 1 (YB-1) is linked to poor prognosis in ERG-negative prostate cancer**

Asmus Heumann^1,2,5^, Özge Kaya^1,5^, Christoph Burdelski^2^, Claudia Hube-Magg^1^, Martina Kluth^1^, Dagmar S. Lang^1^, Ronald Simon^1,*^, Burkhard Beyer^3^, Imke Thederan^3^, Guido Sauter^1^, Jakob R. Izbicki^2^, Andreas M. Luebke^1^, Andrea Hinsch^1^, Frank Jacobsen^1^, Corinna Wittmer^1^, Franziska Büscheck^1^, Doris Höflmayer^1^, Sarah Minner^1^, Maria Christina Tsourlakis^1^, Thorsten Schlomm^3,4^, and Waldemar Wilczak^1^

**Supplementary Table S1.** Association between combined YB-1 staining results and prostate cancer phenotype in ERG-negative cancers.

Abbreviation: tertiary 5 (Tert.5)

**Supplementary Table S2.** Association between combined YB-1 staining results and prostate cancer phenotype in ERG-positive cancers.

Abbreviation: tertiary 5 (Tert.5)

**Supplementary Figure S1.** Prognostic impact of Gleason score and YB1 in ERG negative cancers. a) Impact of classical Gleason score categories (indicated by black dotted lines), and YB-1 negative (red line) or nuclear accumulation positive (blue line) cancers. b-h) Impact of quantitative Gleason score categories defined by percentage of Gleason 4 patterns b) ≤5% Gleason 4, c) 6-10% Gleason4, d) 11-20% Gleason 4, e) 21-30% Gleason 4, f) 31-49 % Gleason 4, g) 50-60% Gleason 4, h) 61-100% Gleason 4 (indicated by black dotted lines), and YB-1 negative (red line) or nuclear accumulation positive (blue line) cancers. Abbreviation: prostate specific antigen (PSA)

**Supplementary Figure S2.** Specificity of the YB-1 (Abcam ab12148) antibody. a) Lack of staining if the YB-1 amtibody was pre-absorbed with a blocking peptide (Abcam ab12411). b) Same tissue spot incubated with YB-1 antibody without blocking peptide.

**
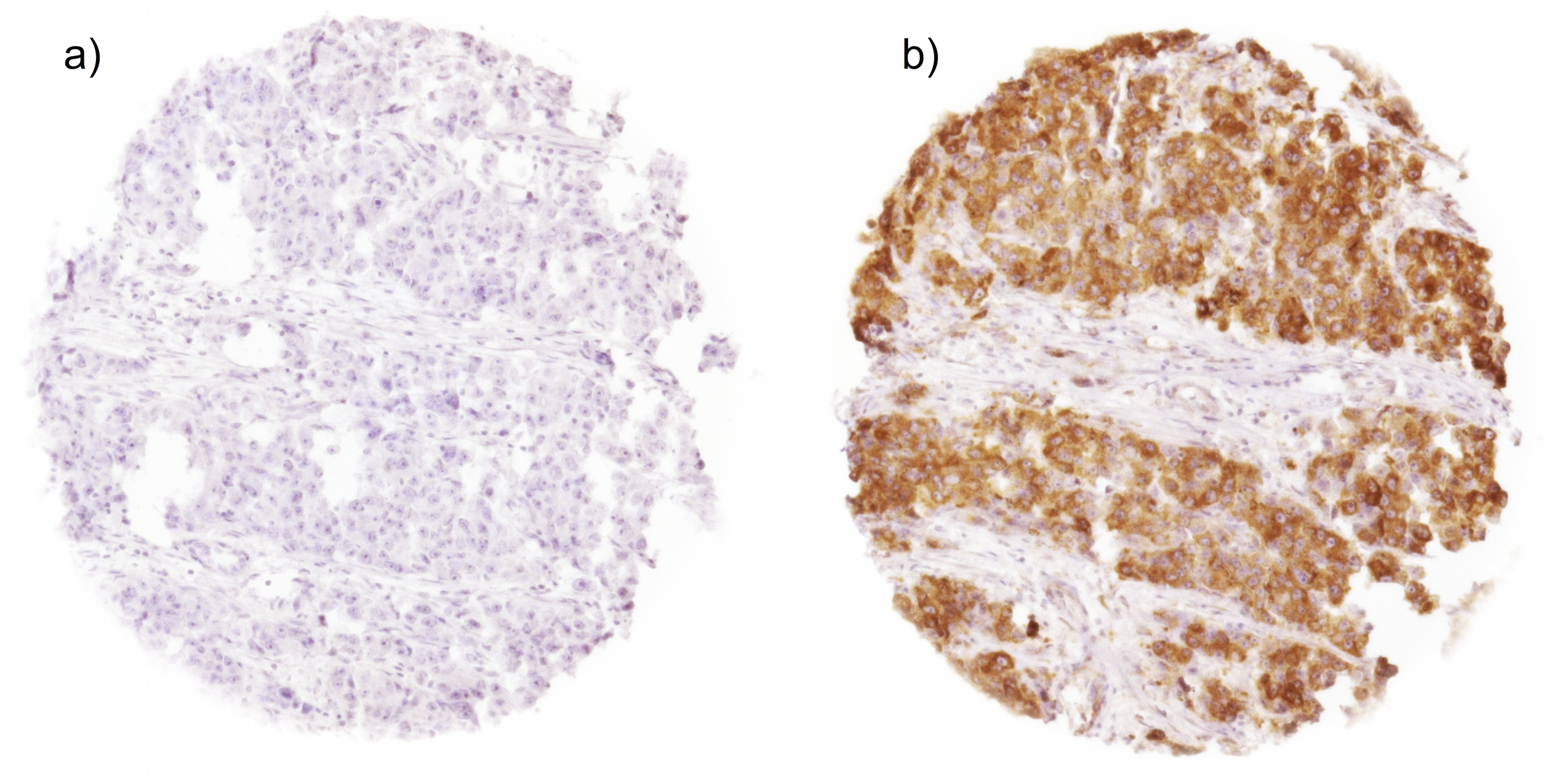
**
